# Establishing a Causal Role for Medial Prefrontal Cortex in Reality Monitoring

**DOI:** 10.3389/fnhum.2020.00106

**Published:** 2020-03-25

**Authors:** Karuna Subramaniam, Hardik Kothare, Leighton B. Hinkley, Phiroz Tarapore, Srikantan S. Nagarajan

**Affiliations:** ^1^Department of Psychiatry, University of California, San Francisco, San Francisco, CA, United States; ^2^UCB/UCSF Graduate Program in Bioengineering, University of California, San Francisco, San Francisco, CA, United States; ^3^Department of Radiology and Biomedical Imaging, University of California, San Francisco, San Francisco, CA, United States; ^4^Department of Neurosurgery, University of California, San Francisco, San Francisco, CA, United States

**Keywords:** reality monitoring, medial prefrontal cortex, repetitive transcranial magnetic stimulation, positive mood, negative mood

## Abstract

Reality monitoring is defined as the ability to distinguish internally self-generated information from externally-derived information. Functional imaging studies have consistently found that the medial prefrontal cortex (mPFC) is a key brain region subserving reality monitoring. This study aimed to determine a causal role for mPFC in reality monitoring using navigated repetitive transcranial magnetic stimulation (nrTMS). In a subject-blinded sham-controlled crossover design, healthy individuals received either active or sham nrTMS targeting mPFC. Active modulation of mPFC using nrTMS at a frequency of 10 Hz, significantly improved identification of both self-generated and externally-derived information during reality monitoring, when compared to sham or baseline. Targeted excitatory modulation of mPFC also improved positive mood, reduced negative mood, and increased overall alertness/arousal. These results establish optimal nrTMS dosing parameters that maximized tolerability/comfort and induced significant neuromodulatory effects in the mPFC target. Importantly, this is a proof-of-concept study that establishes the mPFC as a novel brain target that can be stimulated with nrTMS to causally impact both higher-order reality monitoring and mood.

## Introduction

Reality monitoring is defined as the ability to distinguish internally self-generated information from externally-derived information (Johnson et al., 1993; Keefe et al., 1999; Vinogradov et al., 2008; Subramaniam et al., 2012). Reality monitoring is particularly relevant for patients with schizophrenia who suffer from cardinal impairments of the self, which directly contribute to their psychotic symptoms of delusions and hallucinations (indicating their break with reality; Synofzik et al., 2010; Voss et al., 2017). We and others have recently shown using functional magnetic resonance imaging (fMRI) that the medial prefrontal cortex (mPFC) is a key brain region subserving reality monitoring, which has also been shown to be activated specifically during memory retrieval of self-generated information (i.e., recalling one’s internal thoughts and actions; Vinogradov et al., 2008; Subramaniam et al., 2012, 2016).

Strong convergent evidence across lesion studies, a meta-analysis of voxel-based morphometry (VBM) studies, deep brain stimulation and neuroimaging studies, all strongly suggest that mPFC is also a critical node for mediating mood state (Stefurak et al., 2003; Koenigs et al., 2008; Bora et al., 2012; Downar and Daskalakis, 2013). For example, Downar and Daskalakis (2013) reviewed evidence across distinct and divergent methodologies to show that mPFC mediates mood enhancement in that: (i) lesions to the mPFC induced 80% risk of severe depressive symptomatology (Koenigs et al., 2008); (ii) meta-analysis of VBM studies in Major Depressive Disorder (MDD) revealed the most reductions in volume were found in mPFC (Bora et al., 2012); and (iii) DBS-induced inhibition of mPFC produced intense, dysphoria in a patient with remitted major depression (Stefurak et al., 2003).

We and others have also demonstrated that the mPFC plays a critical function in mediating interactions between mood and higher-order cognition during reality-monitoring (Vinogradov et al., 2008; Subramaniam et al., 2012, 2016, 2017; Subramaniam and Vinogradov, 2013). These prior correlative imaging studies which show that mPFC supports both reality monitoring and mood enhancement underscore the critical need to investigate whether mPFC can *causally* impact reality monitoring and mood. In this study, for the first time, we use neurostimulation in the form of navigated repetitive transcranial magnetic stimulation (nrTMS) to establish the causal role of a novel neural target in the mPFC on impacting higher-order reality monitoring and mood in healthy controls (HC).

rTMS has shown to be a robust neural plasticity-inducing technique that enables alterations of neuronal activity both in stimulated and remote areas *via* trans-synaptic neural plasticity (Pascual-Leone et al., 1996; Hasan et al., 2017). Lower frequencies of TMS at ~1 Hz typically produce inhibitory modulation effects, whereas TMS at more than 5 Hz are thought to generally produce excitatory modulation of the underlying cortical region being targeted (Wassermann and Lisanby, 2001).

Although the first human studies of rTMS took place nearly 30 years ago (George et al., 1995; Pascual-Leone et al., 1996), of all the stimulation targets tested to treat mood disorders and improve mood, the mPFC has received the least attention (Downar and Daskalakis, 2013). This is because the original applications of high-frequency TMS and the most widely used (and only) FDA-approved TMS therapeutic protocol have been applied to the dorsolateral prefrontal cortex (DLPFC), rather than the mPFC (Herbsman et al., 2009). In these therapeutic TMS protocols, patients with MDD are predominantly treated with high-frequency stimulation in the DLPFC of between 10–20 Hz for over 30 min (O’Reardon et al., 2007; Herbsman et al., 2009). Given that the optimal parameters of stimulation are largely unknown particularly concerning the mPFC, in the current study we test shorter protocols in HC that will enable greater affordability and broader transdiagnostic implementation across a range of disorders, such as in patients with mood and psychosis-spectrum disorders.

**Figure 1:**
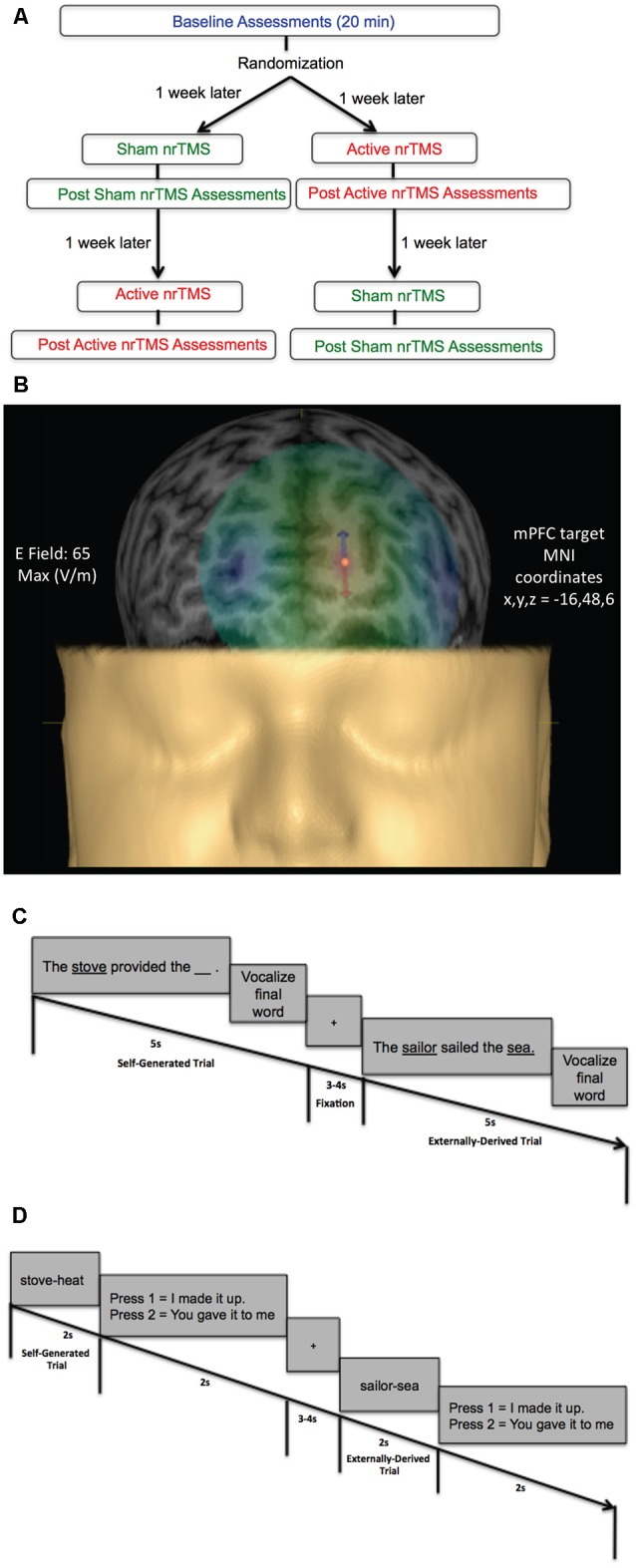
**(A)** Flow chart of randomized subject-blinded sham-controlled crossover navigated repetitive transcranial magnetic stimulation (nrTMS) study trial. Subjects first completed baseline assessments, which included the reality monitoring encoding and retrieval tasks and mood and arousal ratings, for a total duration of 20 min. Subjects were then randomized to either the active nrTMS or sham nrTMS condition first; they completed the first nrTMS session a week after baseline assessments. After nrTMS application (duration = 20 min), subjects immediately performed the post nrTMS reality monitoring assessments and mood/arousal ratings (duration = 20 min). A week later, subjects completed the crossover (i.e., the second) nrTMS session and completed post nrTMS assessments (duration = 20 min) immediately after the nrTMS application. **(B)** A 3-D rendering of one subject’s head model is illustrated as an example, depicting the E-field strength in real-time when applying active high-frequency 10 Hz nrTMS to the medial prefrontal cortex (mPFC) target coordinates (*x*, *y*, *z* = −16, 48, 6), defined by peak reality monitoring activity in our prior neuroimaging studies (Subramaniam et al., 2012; Subramaniam and Vinogradov, 2013). **(C)** Reality monitoring encoding task: participants were presented with noun-verb-noun sentences in which the final word was either left blank for participants to generate themselves (e.g., *The stove provided the* ___ ) or was externally-derived as it was provided by the experimenter (e.g., *The sailor sailed the sea*). For each sentence, participants were told to pay attention to the underlined words and to vocalize the final word of each sentence. **(D)** Reality monitoring retrieval task: participants were randomly presented with the noun pairs from the sentences, and subjects had to identify with a button-press whether the second word was previously self-generated (e.g., stove-heat) or externally-derived (e.g., sailor-sea).

Here we apply high-frequency (10 Hz) nrTMS targeting mPFC to increase its activity (Rabey et al., 2013; Figure 1A). Targeting of mPFC was based on coordinates derived from our prior imaging studies on reality monitoring (Subramaniam et al., 2012; Subramaniam and Vinogradov, 2013; Figure 1B). Our primary hypothesis was that increased excitatory activity induced by high-frequency 10 Hz nrTMS targeting mPFC when compared to baseline and sham would result in improvement of higher-order reality monitoring (Figures 1C,D). Evidence in support of this hypothesis would thus establish for the first time a powerful causal relationship between mPFC activity and reality monitoring. A second hypothesis we tested here was that high-frequency nrTMS targeting mPFC would improve mood state in HC.

A final objective of the present study was to determine sufficient nrTMS dosage in HC, to test whether shorter protocols targeting the mPFC as a novel stimulation site in HC may be effective for improving both higher-order cognition during reality monitoring and mood state. Prior studies have shown that high-frequency 10 Hz stimulation applied in 2 s trains to the lateral PFC is beneficial to cognition (Bentwich et al., 2011; Rabey et al., 2013; Lee et al., 2016). Therefore, consistent with these studies (Bentwich et al., 2011; Rabey et al., 2013; Lee et al., 2016), here, we use similar parameters applied to the mPFC for the first time for a total duration of 20 min, which we hypothesized would yield beneficial mood and cognitive effects during a higher-order reality monitoring task assessed immediately after the nrTMS, but that would also meet safety and tolerability criteria in HC.

## Materials and Methods

### Participants

This study was approved by the Internal Review Board (IRB) at the University of California San Francisco (UCSF) and all research was performed following IRB regulations at UCSF. Participants were evaluated by a clinical psychologist and completed questionnaires to meet the following inclusion criteria. Inclusion criteria for healthy participants were: no psychiatric/neurological disorders, including no personality disorders, no current or history of substance dependence or substance abuse, meets MRI criteria, good general physical health (i.e., no current medical illness or physical injuries), age between 20 and 40 years, right-handed, and English as the first language. All participants (five males, six females, mean age = 31.84, mean education = 18.95) gave written informed consent and then completed the reality monitoring task and mood and alertness/arousal ratings at baseline (Figure 1A). After baseline assessments, participants were randomly assigned in a subject-blinded sham-controlled nrTMS trial to active nrTMS or the sham nrTMS condition first in our subject-blinded crossover design and then assigned to the other nrTMS condition, totaling 33 data test points across all participants (Figure 1A). Thus, in this tightly-controlled longitudinal trial, each participant completed mood and reality-monitoring assessments (of ~20 min duration) at three time points (at baseline, immediately after sham nrTMS and immediately after active nrTMS). Each participant was instructed to maintain as constant a state as possible when compared to the baseline assessments (in terms of the amount of sleep, caffeine, exercise, mood, and alcohol intake). Only the person administering nrTMS was aware of whether each participant received active nrTMS or sham nrTMS and was not allowed to discuss randomization with the subject (for complete details, see “Active and Sham nrTMS” section below). Order of the active and sham nrTMS was counterbalanced across subjects. One participant was unable to tolerate the active nrTMS due to referred pain at the back of her head, and another participant completed the active nrTMS but was unable to complete the sham nrTMS condition due to relocation to another city.

### Reality Monitoring Task

All subjects did a practice reality monitoring task before the reality monitoring task that was conducted at baseline. Subjects then completed the reality monitoring task at baseline and immediately after each active nrTMS and sham nrTMS session. Reality monitoring requires that subjects make higher-order judgments about distinguishing whether the information was previously self-generated or externally-derived. Consistent with our prior studies (Subramaniam et al., 2012, 2016, 2017; Subramaniam and Vinogradov, 2013), subjects completed three runs altogether, with 20 trials per run, totaling 60 trials for each reality monitoring session. As described in previous experiments, the reality monitoring task consisted of an encoding phase and a memory retrieval phase (Subramaniam et al., 2012, 2016, 2017; Subramaniam and Vinogradov, 2013; Figures 1C,D). During encoding, participants were visually presented with semantically constrained sentences with the structure “noun-verb-noun,” presented in blocks of 20 trials per run. On alternating half of the sentences, the final word was either left blank for participants to generate themselves (e.g., *The stove provided the* ___ ) or was externally-derived as it was provided by the experimenter (e.g., *The sailor sailed the sea*). For each sentence, participants were told to pay attention to the underlined words and to vocalize the final word of each sentence. Participants then completed the reality-monitoring retrieval task where they were randomly presented with the noun pairs from the sentences (e.g., *stove-heat*), and had to identify with a button-press whether the second word was previously self-generated or externally-derived (Figures 1C,D). At each time point, the reality monitoring task consisted of different sets of matched semantically constrained sentences, based on our previous studies (Vinogradov et al., 2008; Subramaniam et al., 2012, 2016, 2017; Subramaniam and Vinogradov, 2013). The number of correctly identified self-generated and externally-derived trials was computed for each participant during the retrieval phase.

### Mood and Arousal Ratings

We attained visual analog measures of tolerability and effectiveness of each nrTMS session, as well as mood and arousal/alertness ratings before and after each nrTMS session rated on a visual analog scale from 0 to 9 (i.e., 0 = Not at all; 9 = Very high) at baseline, and also immediately before and after each nrTMS active and sham session. The visual analog scale is a 10 cm line with numbers marked from 0 to 9, but with labels only on 0 (“Not at all”) and 9 (“Vey high”), to minimize subject scale rating bias. The experimenter does not influence subjects’ self-reported ratings since subjects rated their mood and arousal levels on their own without any prompting from the experimenter. Mood and alertness/arousal ratings for each condition are shown in Table 2.

**Table 1:** Reality-monitoring accuracy.

	Self-generated identification	Externally-derived identification	Overall accuracy (*d*-prime)
Baseline	83.00 ± 9.99	82.33 ± 9.17	1.99 ± 0.55
After active 10 Hz rTMS	88.67 ± 7.57	90.00 ± 10.89	2.79 ± 0.90
After sham rTMS	85.56 ± 7.64	83.70 ± 9.35	2.24 ± 0.85

*Repeated-measures ANOVAs reveal significant improvements in d-prime scores, self-generated and externally-derived accuracy after active nrTMS compared to baseline (*p* = 0.007, *p* = 0.04, *p* = 0.002, respectively). Subjects also identified more self-generated and externally-derived information after active nrTMS compared to sham (*p* = 0.04, *p* = 0.03, respectively)*.

**Table 2:** Mood and arousal ratings.

	Positive mood	Negative mood	Alertness/Arousal
Baseline	4.98 ± 1.63	4.30 ± 2.67	5.90 ± 1.66
After active 10 Hz rTMS	5.73 ± 1.20	2.70 ± 2.31	7.10 ± 1.52
After sham rTMS	4.78 ± 1.56	4.67 ± 1.94	5.22 ± 1.48

*Repeated-measures ANOVAs reveal improvements after active nrTMS in negative mood, positive mood and arousal levels compared to baseline (*p* = 0.03, *p* = 0.08, *p* = 0.05, respectively); marginal improvements in negative mood and positive mood after active nrTMS compared to sham (*p* = 0.07, *p* = 0.07, respectively), and significant improvements in arousal levels after active nrTMS compared to sham (*p* = 0.046)*.

### Behavioral Statistical Analyses

Signal detection theoretic *d*-prime analyses for the reality monitoring task were conducted on overall accuracy in identification of word items by calculating the hit rate and the false alarm rate for self-generated and externally-presented items, then converting each measure to *z*-scores, and subtracting the false alarm rate from the hit rate to differentiate sensitivity during accurate performance from response bias. Self-generated and externally-derived accuracy was computed as a percentage of the total number of self-generated and externally-presented trials in each condition (i.e., baseline, sham nrTMS, and active nrTMS) for each subject, and then averaged across all subjects. Repeated-measures ANOVAs with follow-up pairwise comparisons were implemented to examine differences in reality monitoring performance and mood ratings between baseline, active nrTMS, and sham nrTMS conditions. Outliers were defined as values above/below two standard deviations from the mean. We did not find any outliers in the behavioral data. For repeated measures designs, effect sizes are computed using Cohen’s *d* as below:

$$d=\frac{\left|m_{1}-m_{2}\right|}{\sqrt{s_{1}^{2}+s_{2}^{2}-(2rs_{1}s_{2})}}$$

where *r* is the correlation between the two conditions. Mean accuracy in each condition is illustrated for overall *d*-prime accuracy, and for correctly identified self-generated and externally-derived information, averaged across all participants (Table 1).

### Safety and Tolerability of nrTMS

We first pilot tested three HC on four different nrTMS protocols before the start of the study to establish optimal dosage parameters that maximized tolerability/comfort in the present study. We tested each HC using the following nrTMS protocols, counterbalanced for each subject: (i) 20 Hz nrTMS for 2 s trains at 110% of their resting motor threshold (RMT) with an ITI of 28 s; (ii) 10 Hz nrTMS for 4 s trains at 110% of their RMT with an ITI of 26 s; (iii) 10 Hz nrTMS for 2 s trains at 120% of their RMT with an ITI of 28 s; and (iv) 10 Hz nrTMS for 2 s trains at 110% of their RMT with an ITI of 28 s (Supplementary Figure S3). The first protocol was least tolerable and most painful while the fourth protocol yielded highest tolerability ratings, and has also shown therapeutic efficacy in other studies (Bentwich et al., 2011; Rabey et al., 2013; Lee et al., 2016). In the present study, we, therefore, implemented the fourth nrTMS protocol for 20 min to examine its effects in healthy individuals during reality monitoring, given that nrTMS is known to induce long-term potentiation (LTP) in neural activity in stimulated and remote brain areas expected to last beyond and for at least the length of the period of the nrTMS duration (Hasan et al., 2017; i.e., here, we would expect nrTMS effects to last for at least 20 min after the TMS stimulation). Due to prior reports of pain with 20 Hz stimulation from our pilot test sample, we did not use higher frequencies than 10 Hz or theta-burst stimulation, or longer protocols and single train durations (>2 s), which have a higher risk for pain and seizures. The online repetitive nrTMS parameters that we are using have also been deemed safe by the 2009 Consensus Guidelines (Rossi et al., 2009). Safety is defined by the absence of adverse events, such as seizures. As depicted in Rossi et al. (2009), the maximum safe duration at 10 Hz is >5 s. Thus, our exposure of 2 s at 10 Hz is well within the established range.

### Active and Sham nrTMS

After completing baseline assessments, participants were randomly assigned to active nrTMS or sham nrTMS condition first in a subject-blinded crossover design (Figure 1A). Targeting of nrTMS stimulation was carried out based on a high-resolution anatomical T1-weighted MRI scan previously obtained for each subject. The nrTMS was delivered *via* state-of-the-art Nexstim Navigated Brain Stimulation (NBS; Nexstim Oy, Helsinki, Finland). This system integrates the TMS figure-8 coil with a software-based frameless stereotactic navigational system that allows for highly accurate cortical targeting that is individualized for each subject. Nexstim uses in-plane coil geometry to simulate the induced electric field and to make reliable predictions of electromagnetic field strength at the target location of neuronal activation (Petrov et al., 2017). The system calculates, in real-time, the strength of the electric field on the cortical target (Ruohonen and Ilmoniemi, 1999). With the Nexstim system, we were thus able to calculate the electric field strength in real-time as 65 V/m for an individual subject (see Figure 1B) when targeting the specific mPFC MNI coordinates (*x* = −16, *y* = 48, *z* = 6) that were based on our prior functional localization of mPFC peak activity mediating reality monitoring (Subramaniam et al., 2012). An integrated electromyography system allows for online detection of motor evoked potentials that result from TMS. This technology overcomes the problems of targeting and repeatability associated with traditional non-navigated TMS by allowing accurate spatial localization and field-strength calculations. Each participant completed three procedures: (I) MRI to Head Registration, which co-registers the patient’s anatomy to the navigational software; (II) Resting Hand Motor Threshold Determination, which maps out the hand motor area and determines each participant’s individual RMT; and (III) two nrTMS sessions, during which each participant received one active and one sham nrTMS which were separated by a week but conducted at roughly the same time of day for that subject.

*(I) MRI to Head Registration*. The nrTMS session began with aligning the MRI images to the subject’s head *via* the MRI to the Head Registration process. Using the bridge of the nose and the crus of the helix of the ear, the 3D-locations of the landmarks (visible on both the subject’s MRI and the head) were measured using a digitizing pen with an optical tracking system. The optical tracking system used two cameras to triangulate the location in 3D space of infrared reflectors attached to the coil and subject’s head.

*(II) Resting Hand Motor Threshold Determination*. Once the co-registration was completed, subjects completed single-pulse TMS mapping of the hand motor region. The RMT was obtained by delivering single TMS pulses to the left motor cortex hand area. RMT was defined as the minimum stimulation intensity at which motor evoked potentials (thresholded at >50 microvolts) were observed 50% of the time from surface muscle recordings from the first dorsal interosseous muscle of the right hand in half of the trials.

*(III) nrTMS Session*. The target region for the nrTMS stimulation was the mPFC, located using MNI coordinates (*x*, *y*, *z* = −16, 48, 6) based on functional localization of mPFC peak activity mediating reality monitoring in HC, which promisingly also showed peak activity increase (*x*, *y*, *z* = −16, 48, 6) in schizophrenia (SZ) patients after behavioral cognitive training interventions (Subramaniam et al., 2012). During the active nrTMS session, subjects received 800 pulses of 10 Hz nrTMS for 2 s at 110% of their RMT with an ITI of 28 s for a total duration of 20 min. nrTMS at this frequency and duration causes an increase in brain excitability over the stimulated area that lasted for the duration of the nrTMS session (at least 20 min; Rabey et al., 2013). Pulse delivery was software controlled. The nrTMS application and immediate post nrTMS assessments altogether lasted approximately 40 min. Each participant also received sham stimulation in which we used the same nrTMS protocol parameters as the active nrTMS condition to target the same mPFC co-ordinates but with the coil tilted 45 degrees from the scalp. Prior research has shown that this method has been useful for preserving the subject blind (Mennemeier et al., 2009). The sham condition mimics the same noise and muscle twitches as active nrTMS but without inducing a significant magnetic field. Only the person administering nrTMS was aware of whether each participant received active nrTMS or sham nrTMS and was not allowed to discuss randomization with the subject.

## Results

### mPFC Modulation Improves Retrieval Accuracy in Reality Monitoring

**Figure 2:**
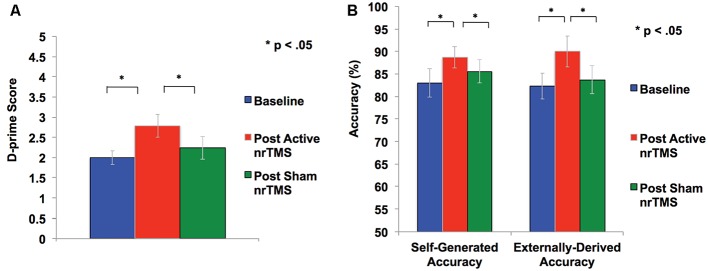
**(A)** Overall accuracy was computed as a *d*-prime score in each condition to differentiate sensitivity from response bias. Repeated-measures ANOVA revealed subjects had significant improvement in *d-prime* statistical scores for overall accurate identification of word items after active rTMS when compared to baseline or sham conditions. **(B)** Self-generated and externally-derived accuracy is illustrated as a percentage of the total number of self-generated and externally-presented trials in each condition (i.e., baseline, sham nrTMS, and active nrTMS), averaged across all subjects. Repeated-measures ANOVA revealed subjects had significant improvement in accurate retrieval of self-generated and externally-presented item-identification after active nrTMS when compared to baseline or sham nrTMS conditions.

A repeated-measures ANOVA showed a significant effect on overall reality monitoring accuracy (*F* = 5.79, *p* = 0.033). Follow-up comparisons indicated that active nrTMS targeting of mPFC at 10 Hz significantly improved reality monitoring performance, indexed by increased d-prime scores for overall accuracy when compared to baseline (*p* = 0.007) and sham conditions (*p* = 0.025; Figure 2A). Large effect sizes were found after active nrTMS targeting mPFC compared to baseline (Cohen’s *d* = 1.2) and sham conditions (Cohen’s *d* = 1.1). Medium to large effect sizes showing improvements in retrieval accuracy after active nrTMS targeting mPFC compared to baseline and sham were observed for both self-generated (Cohen’s *d* = 0.75 and 0.63, respectively) and externally-derived information (Cohen’s *d* = 1.4 and 0.90, respectively; Figure 2B). When compared to baseline, subjects accurately identified significantly more self-generated (*p* = 0.04) and externally-derived information (*p* = 0.002). When compared to the sham condition, subjects also accurately identified significantly more self-generated (*p* = 0.04) and externally-derived information (*p* = 0.03). There were no significant differences in reality monitoring performance between the sham nrTMS and baseline conditions or an order effect in nrTMS condition assignment on reality monitoring performance (all *p*’s > 0.20), indicating that any changes resulting from the active TMS were not due to practice effects and did not result from subjects being un-blinded/aware of the study design. Indeed, before un-blinding at study completion, no participant was aware of the study design.

Only one subject was unable to complete sham TMS, leaving a total of 29 data points for valid test-retest reliability measurements. Intraclass correlation coefficients (ICC) of 0.7 between reality monitoring scores at baseline and after sham nrTMS, showed high test-retest validity and reliability of the reality-monitoring assessments. A sample size of *N* = 10 is required to detect an ICC of 0.7 (Brunelin et al., 2006), as shown in the present study.

### mPFC Modulation Improves Mood

**Figure 3:**
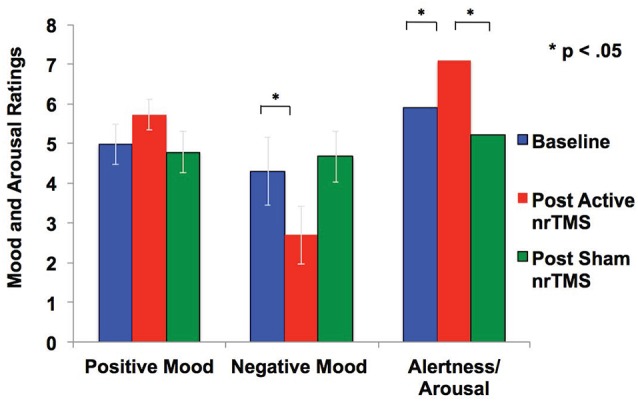
Mood and arousal/alertness in each condition (i.e., baseline, sham nrTMS, and active nrTMS) were rated on a visual analog scale from 0 to 9 (i.e., 0 = Not at all; 9 = Very high). Repeated-measures ANOVA revealed subjects had increased positive mood, reduced negative mood, and increased alertness/arousal levels after active nrTMS when compared to baseline assessments.

After active nrTMS of mPFC, subjects had significant reductions in their negative mood (*p* = 0.03) and marginal improvements in a positive mood (*p* = 0.08) when compared to baseline (Figure 3). After active nrTMS when compared to sham, subjects showed marginal improvement in positive and negative mood states (*p* = 0.07; *p* = 0.07, respectively). They also showed significant improvements in overall alertness/arousal levels after active nrTMS when compared to baseline (*p* = 0.05) and sham conditions (*p* = 0.046). Large effect sizes were found after active nrTMS targeting mPFC in positive and negative mood and arousal levels, compared to baseline (Cohen’s *d* = 0.73, *d* = 0.90, *d* = 0.86, respectively) and compared to sham conditions (Cohen’s *d* = 0.69, *d* = 0.70, *d* = 0.79). There were no significant differences in mood/arousal levels between sham and baseline conditions or an order effect in nrTMS condition assignment on mood/arousal levels (all *p*’s > 0.40).

### Safety and Tolerability of mPFC Modulation With nrTMS

All HC who completed active nrTMS reported that the active nrTMS was fairly highly tolerable and beneficial (6.15 and 6.20, respectively) on a scale of 0–9 (i.e., 0 = Not at all; 9 = Very high). No seizures or other serious adverse events occurred in any participant. Only one subject experienced referred pain at the back of her head (rather than the frontal region) after the first single nrTMS train for a 2 s duration. We ramped down the intensity after every single train in steps (i.e., to varying intensities at 100%, 90% and 80% of RMT); however, this subject still experienced pain even at 80% of RMT. This subject reported no adverse events or side effects beyond the duration of every single train of nrTMS but was eliminated from the study and further analyses.

## Discussion

In the current subject-blinded sham-controlled crossover study, we found that the active high-frequency 10 Hz nrTMS targeting this functionally localized focal peak of mPFC (Supplementary Figure S1) activity when compared to sham and baseline, significantly improved overall reality monitoring in HC, including accurate identification for both self-generated and externally-derived items (Figure 2 and Supplementary Figure S2). When compared to baseline assessments, active nrTMS significantly improved mood and alertness/arousal levels. Together, our results demonstrate for the first time that the mPFC represents a higher-order neural structure that causally mediates interactions between mood and higher-order reality monitoring that involves distinguishing between internal awareness of one’s thoughts and actions from the outside external world. Consistent with our objectives, here we also established the sufficient nrTMS parameters that would yield significant mood and cognitive improvements in HC on our higher-order reality monitoring task but that would also meet safety and tolerability criteria so that these same parameters could then be applied to a larger sample of HC and also be extended to patients with psychiatric disorders in future studies.

We have previously demonstrated in our fMRI studies that mPFC activation correlates with improved reality monitoring not only in HC but also in psychiatric patient populations (i.e., patients with mood and psychotic disorders such as schizophrenia and schizoaffective disorders; Subramaniam et al., 2012, 2016, 2017; Subramaniam and Vinogradov, 2013). Psychosis typically emerges during excessive pruning of excitatory pathways, leading to hypoactive aberrant networks in the PFC (Keshavan et al., 1994; Insel, 2010). Thus, these prior findings are particularly exciting, as they indicate that aberrations in mPFC activity are not immutably fixed even in chronically-ill patients with schizophrenia but that activity can be increased by behavioral interventions such that they became correlated with improvements in reality monitoring and real-world functioning (Subramaniam et al., 2012; Subramaniam and Vinogradov, 2013). In other words, a serious cognitive reality monitoring deficit in patients with psychosis and its underlying neural dysfunction indexed by mPFC hypoactivity can be improved by behavioral interventions and can improve quality of life even in patients with chronic psychosis (Subramaniam et al., 2012). Informed by our promising prior neuroimaging results, the nrTMS target we use here was, therefore, based on functional localization of mPFC activity mediating reality-monitoring in HC (*x*, *y*, *z* co-ordinates = −16, 48, 6), which also showed peak increase after cognitive training interventions in psychosis (*x*, *y*, *z* = −16, 48, 6; Subramaniam et al., 2012; Subramaniam and Vinogradov, 2013; Supplementary Figures S1A,B). Together, our results demonstrate that the same region of mPFC that shows activation in HC, can also be activated to restore reality monitoring in psychosis (Subramaniam et al., 2012), indicating that the same mPFC target can be modulated with nrTMS in HC (Supplementary Figure S1C) as well as in patients with mood and psychotic disorders who suffer from severe reality monitoring impairments. This study, therefore, provides a promising neurobiological basis for a future precision medicine nrTMS approach. In particular, here, we localize the spatial aspects of nrTMS concerning each participant’s neuroanatomy in which we adjust the scalp location of the stimulating magnet to the specific mPFC coordinates in which we observed peak reality monitoring activity to maximally engage these relevant mPFC networks mediating reality monitoring. In doing so, for the first time, we establish the powerful causal linkage of how nrTMS targeting mPFC as a novel neuromodulation target improves mood and reality monitoring in HC, thus providing a basis for future novel therapeutic interventions in psychosis in which reality monitoring is impaired.

The present article also extends our previous findings in which we induced positive, neutral and negative mood states in healthy individuals while they performed fMRI to delineate how different mood states modulated subsequent reality monitoring performance. In these prior studies, we found that a positive mood significantly enhanced reality monitoring task performance in HC subjects *via* mPFC-activity enhancement (Subramaniam et al., 2016, 2017). These previous findings demonstrate that mPFC plays a critical functional role in mood-cognition interactions particularly when people are in positive mood states, which subsequently helped them to improve their reality monitoring performance. Our data are also consistent with a recent study in which 10 Hz nrTMS targeting mPFC was shown to be effective in patients with MDD, significantly reducing depression symptoms in half of the patients (Bakker et al., 2015). Additionally, another recent study in schizophrenia has shown that stimulation of the prefrontal cortex with transcranial direct current stimulation improved emotion identification and general social cognition (Rassovsky et al., 2015). The present results, therefore, validate these prior studies and provide the first neuromodulatory demonstration that the mPFC, in particular, causally mediates mood-cognition interactions in that high-frequency stimulation targeting mPFC not only significantly reduced negative mood and improved positive mood but also induced significantly better reality monitoring performance when compared to baseline assessments.

The causal mechanisms as to precisely how high-frequency nrTMS targeting mPFC enhances mood and reality monitoring performance remain to be understood. We do not know the direction of causality, i.e., whether nrTMS first improved mood and overall alertness, which subsequently enhanced reality monitoring, or whether nrTMS improved information encoding and memory retrieval to enable better self-awareness, which then enabled people to attend and improve their mood and alertness. Future neuroimaging studies in which we specifically assay mPFC activity before and after TMS will be required to delineate precisely how mPFC stimulation modulates interactions between positive mood and reality monitoring accuracy.

We did find that mPFC stimulation improved accurate memory retrieval of both self-generated and externally-derived information, contributing to significant improvement in overall reality monitoring performance. We know that TMS propagates trans-synaptically, and that stimulation of mPFC has direct anatomical and functional connections to the DLPFC (Cho and Strafella, 2009; Unschuld et al., 2014), that is consistently activated during external working memory (Curtis and D’Esposito, 2003; Karlsgodt et al., 2009; Haut et al., 2010; Subramaniam et al., 2014; Kumar et al., 2017). The original and most widely used applications of high-frequency TMS have been applied to DLPFC, which is strongly interconnected to mPFC (Herbsman et al., 2009). These findings suggest that high-frequency mPFC stimulation likely increased DLPFC activation which facilitated encoding and memory recall of externally-derived information through trans-synaptic LTP (Rabey et al., 2013; Hasan et al., 2017). LTP is observed in the enhancement of inter-neuronal signal transmission that outlasts the stimulation. LTP forms the basis of cellular mechanisms subserving learning and memory, based on the fact that memories are encoded by changes in synaptic strength (Bliss and Collingridge, 1993; Rabey et al., 2013; Hasan et al., 2017). An abundant of high-frequency magnetic stimulation studies done in both humans and animals have demonstrated that TMS is a prevalent plasticity-inducing technique, which in humans is thought to be dependent on short-term changes of synaptic efficacy as well as on the activity of glutamate receptors and calcium signaling critical for overall learning (i.e., the successful encoding of information) and memory retrieval (Huang et al., 2007; Ziemann et al., 2008; Hasan et al., 2017).

Although no participant experienced pain in the targeted frontal site, it must be noted that there are likely some subjects who have anatomic variations in the locations of their trigeminal nerve bundles that render them unable to tolerate stimulation, as was the case with the one subject who experienced referred pain at the back of her head. The benefit of nrTMS is that stimulation can be paused immediately for such subjects, unlike pharmaceutical medications that require longer durations and half-lives to be taken up by the whole brain and body, and which consequently result in much worse and long-lasting side-effects. By contrast, the nrTMS dosage can be modified in real-time to optimize patient comfort and ensure tolerability. In the most sensitive patients for whom no physiologically meaningful dose is tolerable, the subject may withdraw from treatment without having to experience protracted discomfort.

Further dose-response studies will be needed to examine the duration and generalizability effects of nrTMS neuromodulation targeting mPFC. In future studies, we will use magnetoencephalography imaging (MEGI) before and after nrTMS to investigate the neural plasticity of how cortical networks are reconfigured after nrTMS-induced mPFC modulation. Improvements in reality monitoring that are related to enhanced mPFC activity will confirm the necessary/causal role of mPFC in the effects of stimulation in these future studies.

## Limitations

Despite having repeated-measures of 29 data points altogether at pre-and post-treatment, the main limitation is the small sample size. As is stated in our RFA MH-18-704 grant that supports this proof-of-concept study, studies during the R61 phase are required to focus on a preliminary evaluation of a treatment effect on manipulating a target, and are required to be pilot studies that should not be powered as strong tests of clinical efficacy, but should rather test the preliminary impact of target engagement on a clinical or cognitive outcome (i.e., in this case, our dependent variables are reality-monitoring performance and mood). To attain a power of 0.7 our sample size of *N* = 10 requires an effect size of Cohen’s *d* = 0.75 to attain a significant treatment effect for repeated-measures designs, thus providing the go-decision criteria for the main findings of this study. Findings must be interpreted with caution for results that show effect sizes that are below a Cohen’s *d* of 0.75. Future studies will involve larger efficacy trials, in which we will test whether the same effects are observed with a larger sample size, including multiple repeated sessions per participant using the same protocol and assessments of multiple covariates.

## Conclusion

In sum, this is a tightly-controlled study, with each subject completing assessments at three time points, that provides the first direct evidence to establish the mPFC as a novel neural target that can be modulated with nrTMS to causally impact both mood and higher-order reality monitoring (Subramaniam et al., 2018). Given that the order of sham nrTMS and active nrTMS were counterbalanced across subjects, and we did not find any order effects, our results cannot be attributed to practice effects on the task. These pioneering findings thus shed new light on applications of nrTMS targeting the mPFC with several far-reaching implications. First, excitatory modulation of mPFC as a novel target in HC can be extended to patients with mood and psychosis-spectrum disorders in which reality monitoring is impaired, particularly in light of our prior neuroimaging findings that the same mPFC neural target is recruited in both HC and patients with psychosis-spectrum disorders (Subramaniam et al., 2012, 2017; Subramaniam and Vinogradov, 2013; Supplementary Figures S1A,B). Second, this study also establishes the single-session nrTMS protocol parameters that meet safety and tolerability criteria and that are sufficient to induce significant neuromodulatory effects in this target. Finally, with our immediate post-stimulation assessments we can maximize LTP effects after only a single session of nrTMS. This may prove highly beneficial for future MEGI studies that explore the timing and duration of cortical network oscillation reconfigurations and plasticity induced by nrTMS. In conclusion, these results provide a neurobiological basis for mPFC to be used as a potential transdiagnostic target for future precision medicine nrTMS therapeutic interventions that aim to improve mood and reality monitoring across disorders.

## Data Availability Statement

All datasets generated for this study are included in the article/Supplementary Material.

## Ethics Statement

The studies involving human participants were reviewed and approved by UCSF IRB approval: 10-02932. The patients/participants provided their written informed consent to participate in this study.

## Author Contributions

KS recruited all the subjects and designed the reality monitoring experiment, acquired, analyzed and interpreted all the data, and wrote and edited the manuscript. HK helped with subject recruitment and acquisition of the nrTMS data. LH provided advice on the acquisition of reality monitoring data. PT conducted the nrTMS sessions on all subjects, and edited the manuscript. SN edited the manuscript and provided advice on the overall acquisition, analyses, and interpretation of all the data.

## Conflict of Interest

The authors declare that the research was conducted in the absence of any commercial or financial relationships that could be construed as a potential conflict of interest.
