# Supplementary material for: Establishing a Causal Role for Medial Prefrontal Cortex in Reality Monitoring
Source: Front Hum Neurosci. 2020 Mar 25;14:106. doi: 10.3389/fnhum.2020.00106 (PMC7109326; doi:10.3389/fnhum.2020.00106)
Supplement: Supplementary file 1 [file Data_Sheet_1.PDF]

Supplementary Figure S1.

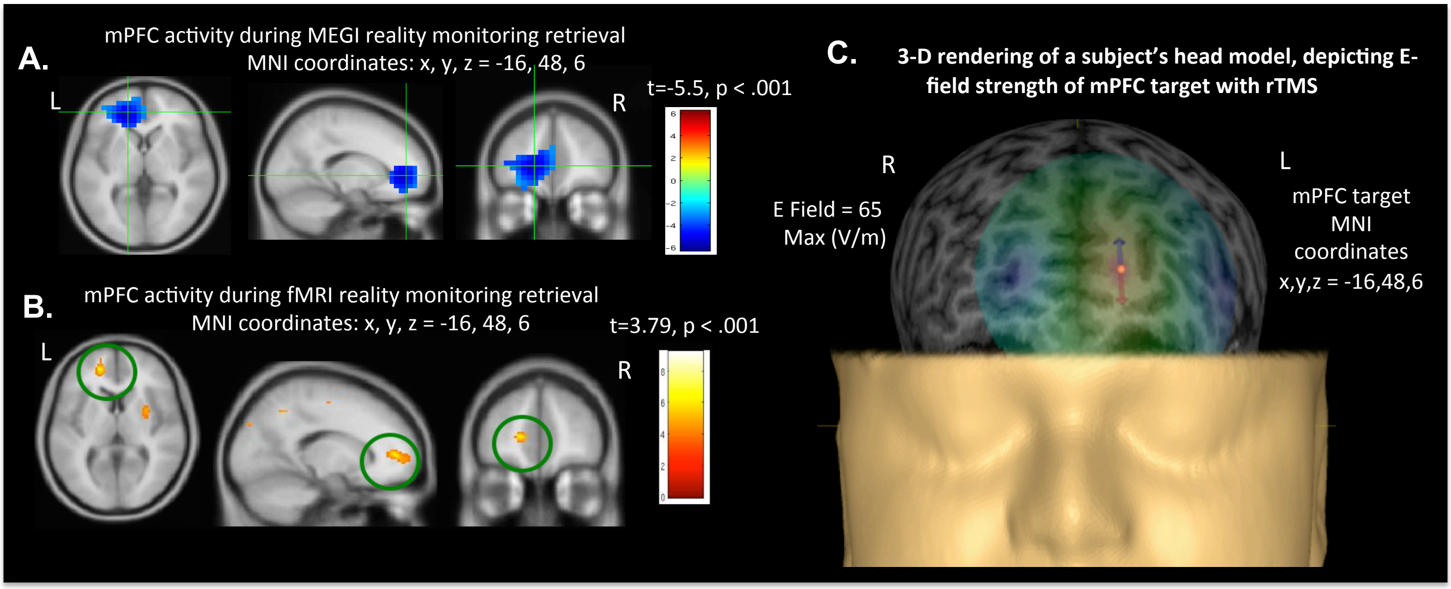

Supplementary Figure S1. A. Axial, sagittal and coronal brain sections showing MEGI changes in beta (12-30Hz) oscillatory power in HC (N=15), indicating significant increases in mPFC activity during reality monitoring self-generated retrieval within a specific time window between 300 to 500ms after stimulus word-pair onset, indicating initial self-awareness of one's own thoughts (Subramaniam et al., 2019). **B.** Axial, sagittal and coronal brain sections showing fMRI changes in mPFC activity increases during reality monitoring self-generated retrieval in patients with schizophrenia (N=15) after patients completed 16 weeks of a behavioral cognitive training intervention compared to baseline, with peak activity in same mPFC region that is activated in HC (Subramaniam et al., 2012). **A and B.** All images are cluster corrected (>100 contiguous voxels) on statistical maps thresholded at $p < 0.001$, centered at mPFC activity peak (x,y,z = -16,48,6). **C.** A 3-D rendering of one subject's head model is illustrated as an example, depicting the E-field strength in real-time when applying active high frequency 10Hz rTMS to the mPFC. This image indicates that the same mPFC target coordinates (x,y,z = -16,48,6) defined by peak reality monitoring activity in our prior neuroimaging studies (**S1A and S1B**), can be targeted and modulated with rTMS.

Supplementary Figure S2

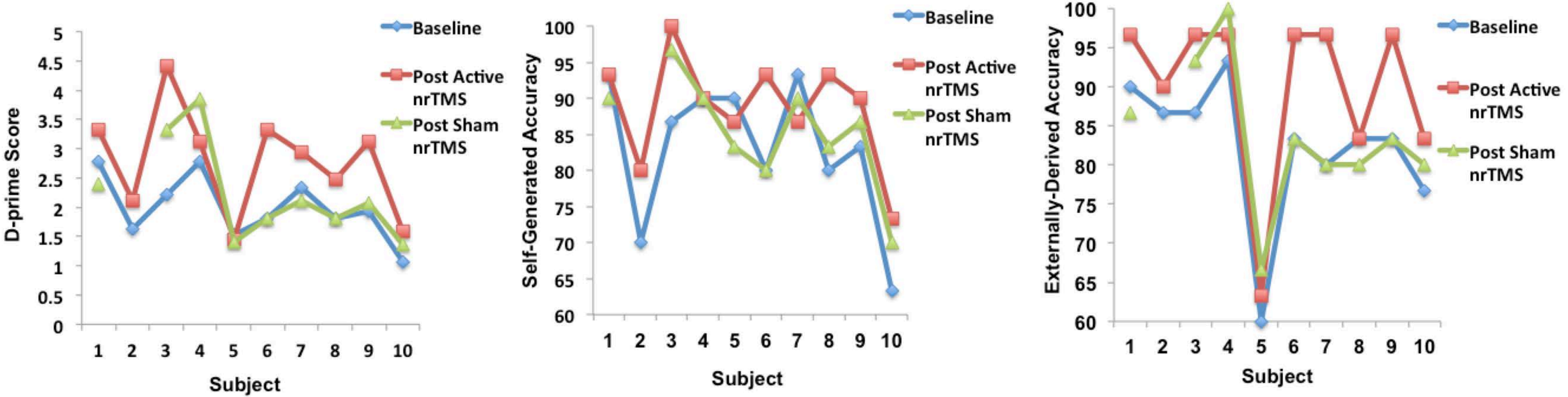

Supplementary Figure S2. Individual subject accuracy data is shown for all 3 conditions: Baseline, Post Sham nrTMS and Post Active nrTMS

Supplementary Figure S3

nrTMS Protocol Pilot Testing Prior to Current Study	Subject Ratings of Tolerability	Subject Ratings of Effectiveness
(i) 20 Hz for 2s at 110% RMT and ITI of 28s	4.33	4.00
(ii) 10 Hz for 4s at 110% RMT and ITI of 26s	6.33	5.33
(iii) 10 Hz for 2s at 120% RMT and ITI of 28s	5.33	5.00
(iv) 10 Hz for 2s at 110% RMT and ITI of 28s	7.00	6.67

Supplementary Figure S3 illustrates the mean subject ratings of tolerability and effectiveness of each TMS protocol during pilot testing prior to the current study in order to determine the optimal TMS dosage parameters for the present study. Based on these results, the fourth protocol yielded highest tolerability and effectiveness, and was thus selected for the current study.
